# Machine‐learning algorithms in screening for type 2 diabetes mellitus: Data from Fasa Adults Cohort Study

**DOI:** 10.1002/edm2.472

**Published:** 2024-02-27

**Authors:** Hanieh Karmand, Aref Andishgar, Reza Tabrizi, Alireza Sadeghi, Babak Pezeshki, Mahdi Ravankhah, Erfan Taherifard, Fariba Ahmadizar

**Affiliations:** ^1^ Student Research Committee, School of Medicine Fasa University of Medical Sciences Fasa Iran; ^2^ USERN Office Fasa University of Medical Sciences Fasa Iran; ^3^ Noncommunicable Diseases Research Center Fasa University of Medical Science Fasa Iran; ^4^ Student Research Committee, School of Medicine Shiraz University of Medical Sciences Shiraz Iran; ^5^ Health Policy Research Center, School of Medicine Shiraz University of Medical Sciences Shiraz Iran; ^6^ Clinical Research Development Unit, Valiasr Hospital Fasa University of Medical Sciences Fasa Iran; ^7^ Data Science and Biostatistics Department Julius Global Health Utrecht The Netherlands

**Keywords:** machine learning, screening, type 2 diabetes mellitus

## Abstract

**Introduction:**

The application of machine learning (ML) is increasingly growing in biomedical sciences. This study aimed to evaluate factors associated with type 2 diabetes mellitus (T2DM) and compare the performance of ML methods in identifying individuals with the disease in an Iranian setting.

**Methods:**

Using the baseline data from Fasa Adult Cohort Study (FACS) and in a sex‐stratified manner, we studied factors associated with T2DM by applying seven different ML methods including Logistic Regression (LR), Support Vector Machine (SVM), Random Forest (RF), K‐Nearest Neighbours (KNN), Gradient Boosting Machine (GBM), Extreme Gradient Boosting (XGB) and Bagging classifier (BAG). We further compared the performance of these methods; for each algorithm, accuracy, precision, sensitivity, specificity, F1 score, and Area Under Curve (AUC) were calculated.

**Results:**

10,112 participants were recruited between 2014 and 2016, of whom 1246 had T2DM at baseline. 4566 (45%) participants were males, aged between 35 and 70 years. For males, age, sugar consumption, and history of hospitalization were the most weighted variables regarding their importance in screening for T2DM using the GBM model, respectively; these variables were sugar consumption, urine blood, and age for females. GBM outperformed other models for both males and females with AUC of 0.75 (0.69–0.82) and 0.76 (0.71–0.80), and F1 score of 0.33 (0.27–0.39) and 0.42 (0.38–0.46), respectively. GBM also showed a sensitivity of 0.24 (0.19–0.29) and a specificity of 0.98 (0.96–1.0) in males and a sensitivity of 0.38 (0.34–0.42) and specificity of 0.92 (0.89–0.95) in females. Notably, close performance characteristics were detected among other ML models.

**Conclusions:**

GBM model might achieve better performance in screening for T2DM in a south Iranian population.

## Graphical abstract

Gradient Boosting Machine model might achieve better performance in screening for T2DM in a south Iranian population.

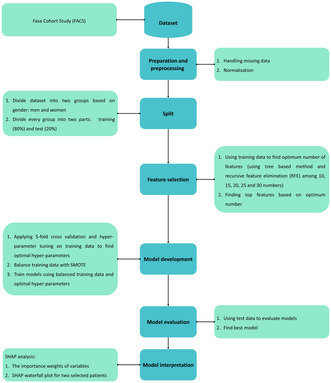

## 1 INTRODUCTION

About 9% of the global population (463 million) suffer from type 2 diabetes mellitus (T2DM). If current trends persist, this number is expected to increase to 10% or roughly 700 million people by 2045.^1^, ^2^ On a global scale, T2DM was responsible for approximately 5 million adult deaths in 2017.^2^ The disease comes with several complications, entangling the patients, their families and public health systems by lowering the quality of life and life expectancy, placing several financial burdens and causing several potentially life‐threatening complications.^3^ The incidence of complications arising from T2DM is considerable, as research indicates that more than half and a quarter of individuals diagnosed with T2DM experience micro and macrovascular complications, respectively.^4^ A 2019 meta‐analysis showed that almost 33%, 38%, 36% and 43% of Iranian patients with T2DM suffer from cardiovascular diseases, neuropathy, retinopathy and nephropathy, respectively.^5^

The timely diagnosis of T2DM is paramount, as it is a chronic disease that can lead to various complications. Early interventions are vital in minimizing the risk of further complications and mitigating the numerous challenges associated with this disease.^6^, ^7^ However, about half of the global T2DM cases are believed to go undiagnosed.^2^ Employing precise screening programs can aid health systems in avoiding overload, organizing their budget, and optimizing care. Hence, prioritizing the development of robust screening and diagnostic strategies is crucial.

Typically, T2DM is diagnosed through direct patient‐physician interaction and requires paraclinical evaluations. Regarding achieving desirable outcomes, preventive programs must maintain their intensity and uptake by ensuring that invitees are covered and willing to accept invitations.^6^ Achieving this goal can be challenging, particularly in settings with limited resources. As a result, implementing low‐cost strategies provided by modern technologies may prove to be a valuable approach, particularly for underserved populations.

To improve the backbone of the evidence on the application of machine learning (ML) in health data, we designed a study employing seven state‐of‐the‐art ML algorithms to evaluate the factors associated with prevalent T2DM in a sex‐stratified analysis and to estimate their performances in screening these patients from the Fasa Adults Cohort Study (FACS). These algorithms included logistic regression (LR), support vector machine (SVM), random forest (RF), K‐nearest neighbours (KNN), gradient boosting machine (GBM), extreme gradient boosting (XGB) and bagging classifier (BAG). The accuracy, precision, sensitivity, specificity, F1 score and area under curve (AUC) were estimated for each model and their performances were compared.

## 2 METHODS

### 2.1 Data sources

**FIGURE 1:**
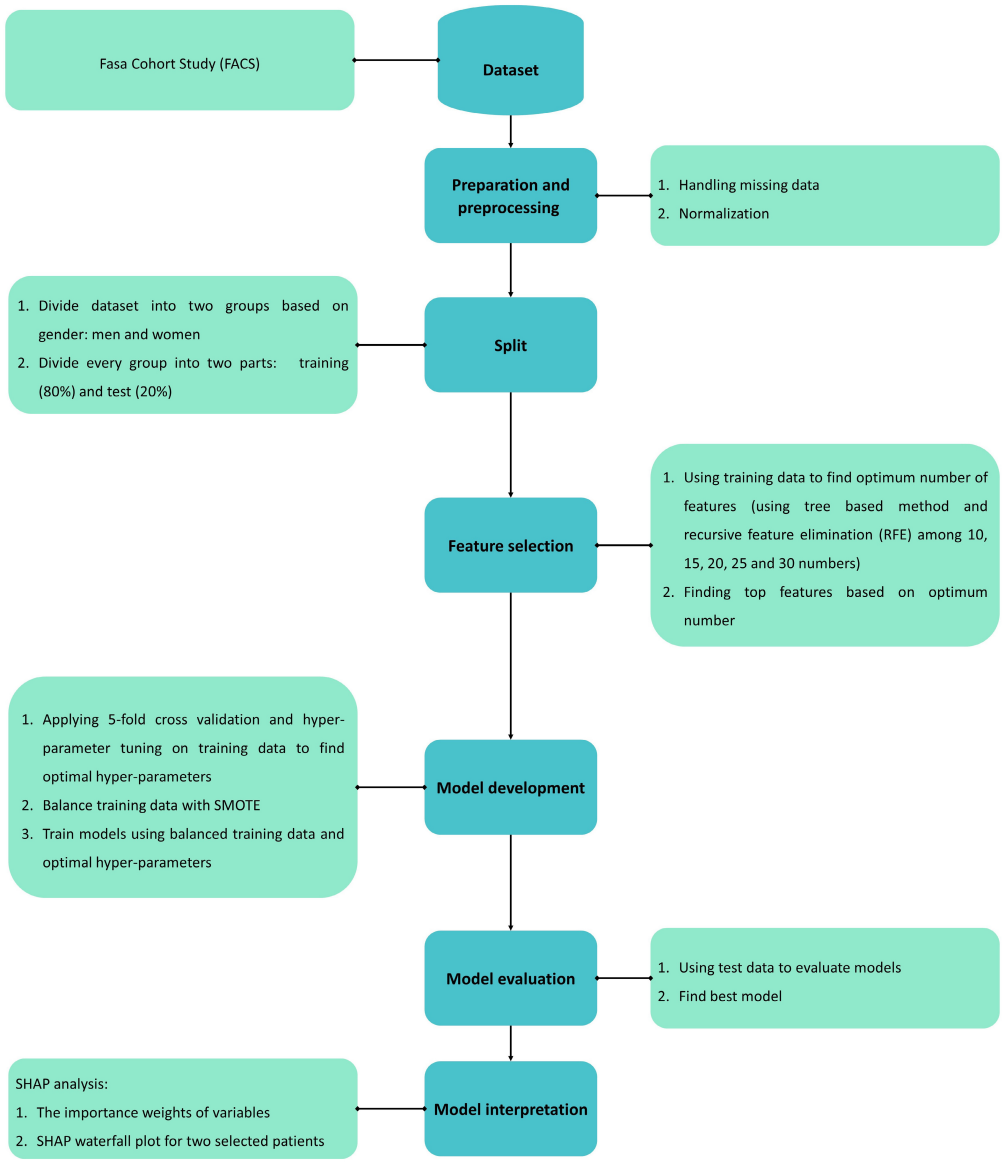
Selection process and workflow.

This is a cross‐sectional (analytical‐descriptive) research based on the baseline FACS data. FACS was developed to assess the risk factors predisposing residents of the Fasa rural region to non‐communicable diseases. In an area where the majority of residents live in rural settings, the enrollment for FACS commenced in October 2014 and concluded in September 2016. Fasa, with a population of approximately 250,000, is located in the Fars province in southwest Iran. The cohort research was carried out in Sheshdeh and Qarabolagh districts of Fasa, a rural region with 41,000 residents. The target population for the cohort included individuals aged between 35 and 70 years who were of Iranian nationality, had been in the area for at least 1 year and capable of effective communication. The FACS was executed using a census method. Within Sheshdeh and Qarabolagh, there were a total of 11,097 individuals in the specified age range, and out of those, 10,622 met the additional eligibility criteria, all of whom were invited to participate in the study. With a participation rate of 95.2%, 10,118 were finally involved in the FACS study. More in‐depth information regarding the objective of the FACS, its methodology, and the sampling region can be found elsewhere.^8^, ^9^ In this study, participants with incomplete data regarding the status of T2DM were not considered. Figure 1 shows the summary of selection process and workflow of the current study.

### 2.2 Data preparation and preprocessing

Variables with missing data were rather prevalent. Analyses that neglect missing data can potentially create biased conclusions. For missing data, we used multiple imputations. Variables with less than 10% of missing values were included in the analysis. The continuous variables were scaled and the variables with more than two categories were transformed into dummy variables.

### 2.3 Primary outcome

The classifier variable for this study was T2DM, dividing individuals into two groups: those with and those without T2DM.^10^ Individuals were categorized as having T2DM if they reported a history of physician diagnosis or if they had been prescribed anti‐diabetic medications.

### 2.4 Splitting data

A sex‐stratified analysis was undertaken, involving separate analyses for males and females. This approach was adopted due to potential variations in risk factors for T2DM between the two sexes. Most variables were shared between males and females; sex‐specific variables were eliminated for each group. Table S1 displays the shared variables and Table S2 shows the specific variables for each gender.

Each group was partitioned into two subsets: Training (80%) and test (20%). Training set was used for feature selection, hyper‐parameter tuning, 5‐fold cross‐validation and data training. The test set was used for final evaluation and internal validation of the ML models.

### 2.5 Feature selection

Feature selection methods were employed to achieve effective data reduction. This is useful for finding accurate data models. There are three types of feature selection: wrapper, filter, and embedded methods.^11^ This study used a wrapper method integrating a tree‐based ML model and recursive feature elimination (RFE). RFE was used to train an RF machine to pick features by iteratively training an ML model and then eliminating the lowest‐ranking features. Initially, laboratory and non‐laboratory variables were chosen based on the gender of each group, existing literature, and FACS data. Consequently, 154 variables for men and 161 variables for women were selected.

**FIGURE 2:**
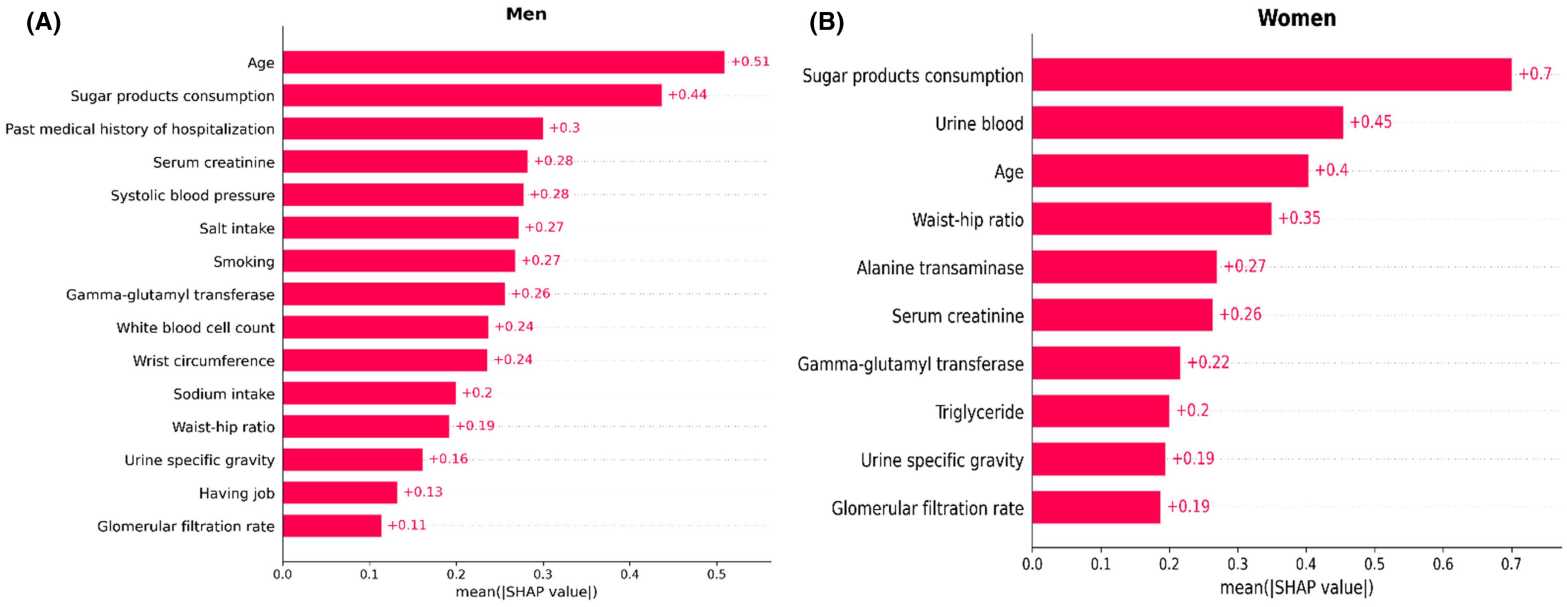
(A, B) The SHAP values of the top features in the GBM model for identification of T2DM. GBM, gradient boosting machine; T2DM, type 2 diabetes mellitus.

Then, RFE and RF were implemented to determine the optimal number of features between 10, 15, 20, 25, and 30. For men, a subset of 15 features demonstrated the highest accuracy, while for women, the optimal subset comprised 10 variables. Further, RFE and RF were employed to identify the most significant features. The top 15 selected features for males included age, past medical history of hospitalization, job status, systolic blood pressure (SBP), waist circumference (WC), waist‐hip ratio (WHR), smoking, white blood cell count (WBC), serum creatinine, gamma‐glutamyl transferase (GGT), urine specific gravity, sodium intake, glomerular filtration rate, sugar products consumption and salt intake (Figure 2A). The top 10 selected features for females consisted of age, WHR, serum creatinine, triglyceride, alanine transaminase (ALT), GGT, urine specific gravity, urine blood, glomerular filtration rate, and sugar products consumption (Figure 2B).

Table 1 presents a descriptive analysis of the selected features. Statistical analyses, including independent *t*‐test, chi‐squared test, and Mann–Whitney‐*U* test, were employed where appropriate. A *p*‐value less than .05 was considered statistically significant. SPSS version 18 (IBM Corp., Armonk, N.Y., USA) was used to analyse the data.

**TABLE 1:** Overview of characteristics of the enrolled participants, including the top 15 important features, categorized by T2DM (*N* = 10,112).

Variables	Type 2 diabetes mellitus		*p*‐value
	No (*N* = 8866)	Yes (*N* = 1246)	
Age, years	47.93 ± 9.46	53.61 ± 8.81	<.001^a^
Sex			
Male	4212 (92.3)	354 (7.7)	<.001^b^
Female	4654 (83.6)	892 (16.4)	
Smoking, yes	1812 (93.3)	130 (6.7)	<.001^b^
Systolic blood pressure, mmHg	110 [92, 128]	117 [98, 136]	<.001^c^
Waist‐hip ratio	0.92 ± 0.06	0.96 ± 0.06	<.001^a^
Wrist circumference, cm	16.71 ± 1.33	16.80 ± 1.38	.029
White blood cell count, ×10^9^/L	6.43 ± 1.69	6.70 ± 1.83	<.001^a^
Triglyceride, mg/dL	129.02 ± 79.63	152.12 ± 97.26	<.001^a^
ALT, U/L	23.17 ± 14.28	25.35 ± 14.78	<.001^a^
GGT, U/L	22.18 ± 19.78	27.55 ± 29.50	<.001^a^
Serum creatinine, mg/dL	0.98 ± 0.18	0.99 ± 0.19	.054^a^
GFR, mL/min/1.73 m^2^	76.44 ± 11.38	70.08 ± 10.94	<.001^a^
Urine specific gravity	1.0205 ± 0.0157	1.0206 ± 0.008	.843^a^
Urine blood, yes	2647 (90.8)	282 (9.2)	<.001^b^
Salt intake, g/day	4.10 ± 2.65	3.69 ± 2.34	<.001^a^
Sugar products consumption, g/day	58.51 ± 59.04	35.44 ± 46.20	<.001^a^
Sodium intake, mg/day	4778 ± 1990	4639 ± 1951	.021^a^
Past medical history of hospitalization, yes	2663 (91.7)	242 (8.3)	<.001^b^
Having job, yes	4686 (92.1)	403 (7.9)	<.001^b^

*Note*: Data are presented as mean ± SD, median [IQR], and number (%).

Abbreviations: ALT, alanine transaminase; GFR, glomerular filtration rate; GGT, gamma‐glutamyl transferase.

^a^ Independent samples test.

^b^ Chi‐squared.

^c^ Mann–Whitney Test.

### 2.6 Machine learning algorithms

Seven supervised ML algorithms, including LR, SVM, RF, KNN, GBM, XGB, and BAG were utilized. The implementation of all ML algorithms was performed using Anaconda (Version 4.12.0) on the Jupyter Notebook Platform (Version 3.3.2). The ML algorithms were run using the Scikit‐Learn Module (Version 1.1.3).

### 2.7 Model development

**FIGURE 3:**
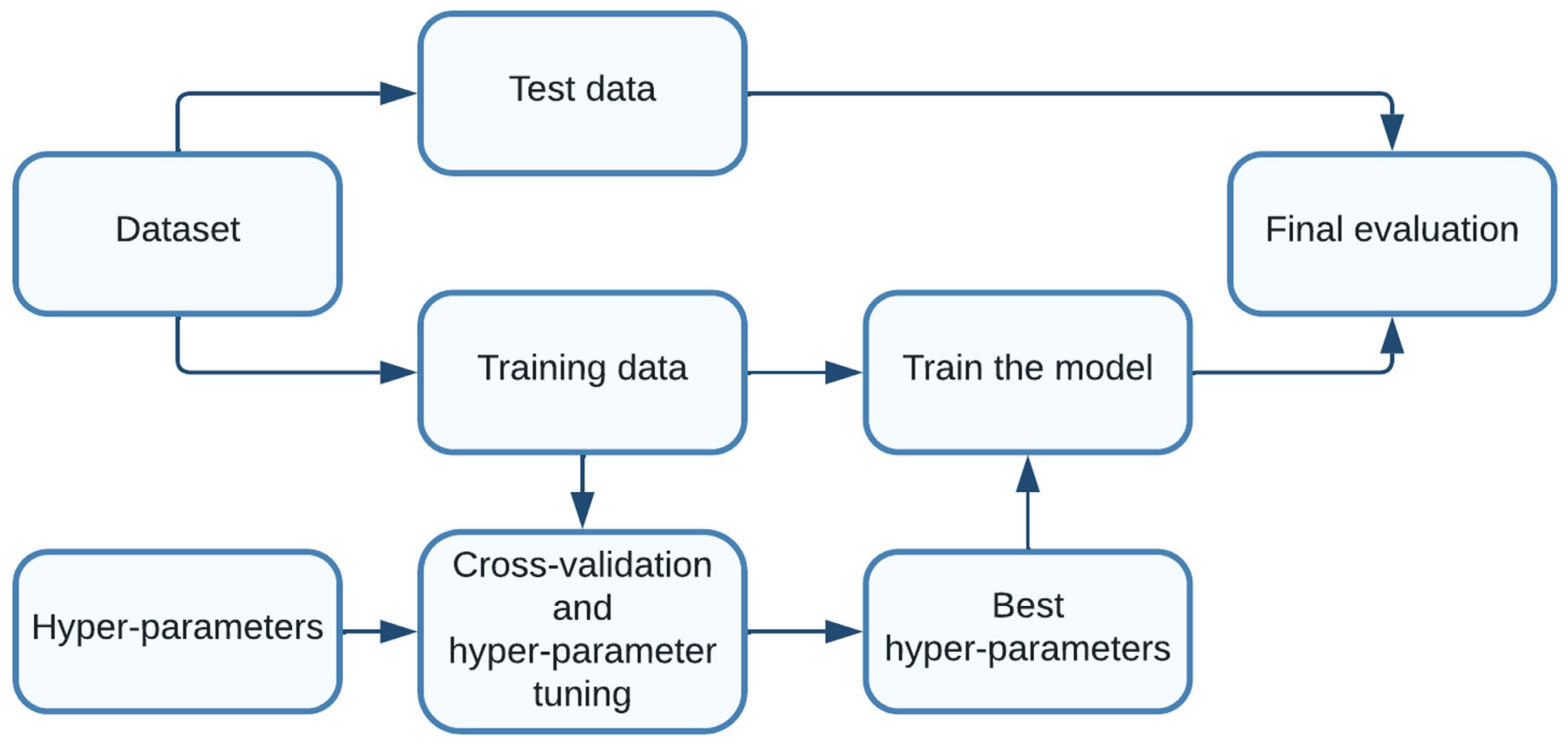
The process of model development with a combination of the hold out method, the 5‐fold cross‐validation method, and the hyper‐parameter tuning.

Initially, the training data underwent 5‐fold cross‐validation and hyper‐parameter tuning to identify the optimal hyper‐parameters. In the 5‐fold technique, the entire training data were partitioned into five equal parts, with each part serving as validation data in turn, being trained itself and its accuracy was recorded. The process was repeated for each part, and the average of all five accuracies was calculated. Subsequently, the accuracy of each ML model was adjusted by modifying its hyper‐parameters. The hyper‐parameter tuning technique involved testing various combinations to discover the optimal set of hyper‐parameters (Figure 3; Tables S3 and S4).^12^

In the second step, over‐sampling was used to balance the values of the outcome, and data with T2DM outcomes were acquired. One of the best over‐sampling approaches is the Synthetic Minority Over‐sampling Technology (SMOTE). Rather than employing replacement, this strategy oversamples the minority class by producing synthetic instances. It selects samples from the minority class and generates synthetic samples along the same line segment, connecting some or all of the minority class's k nearest neighbours.^13^ In this context, participants with T2DM constituted the minority class. SMOTE was applied to generate 3086 instances for men to balance those with and without T2DM and 3010 instances for women to equalize females with and without T2DM. Subsequently, ML algorithms were trained using the balanced training data and optimal hyper‐parameters.

### 2.8 Model evaluation

**FIGURE 4:**
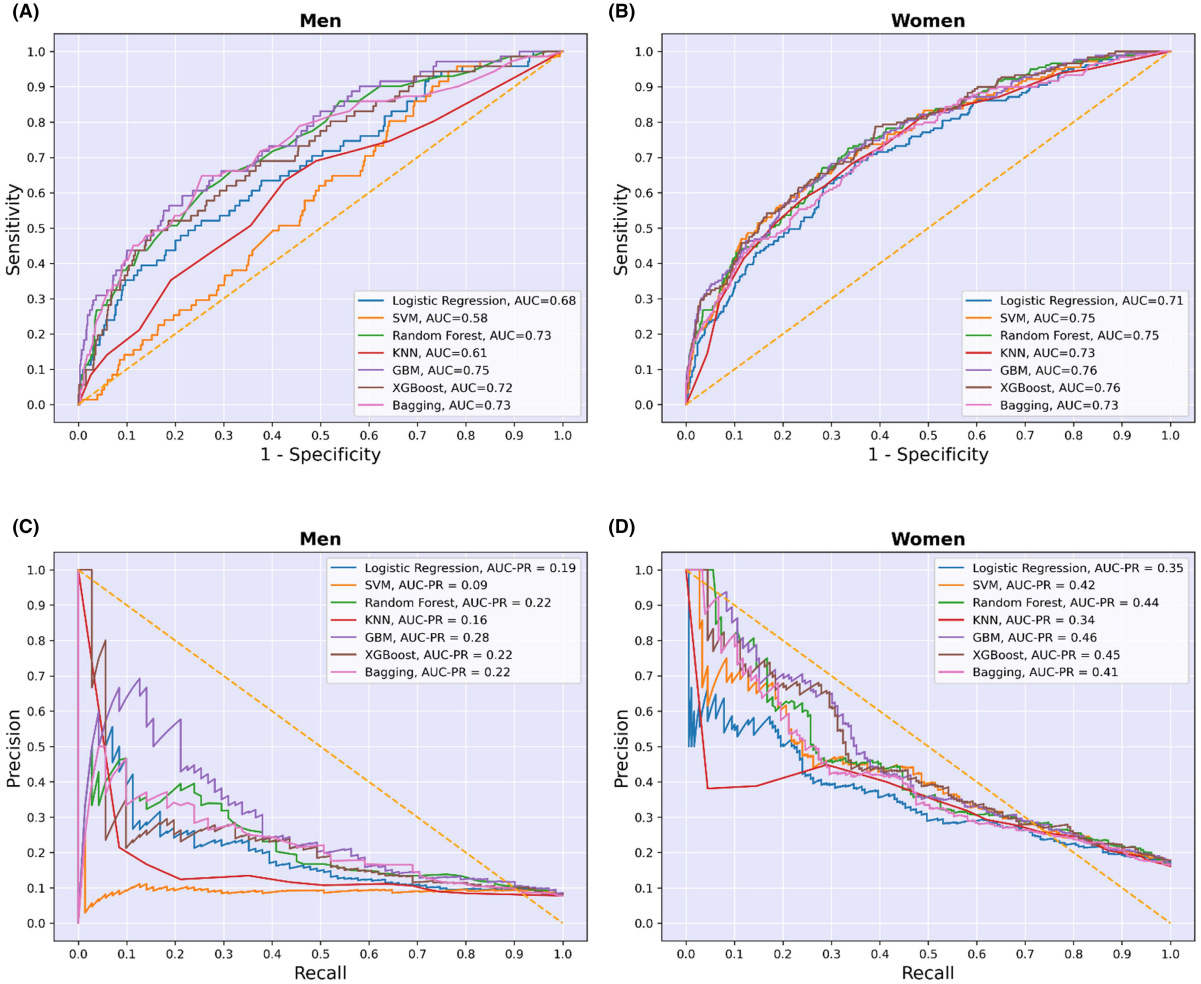
(A, B) Receiver operating characteristic curves of the seven models for men and women. (C, D) Precision‐recall curves of the seven models for men and women.

The trained ML algorithms were applied to the test data for each sex‐stratified group to assess and compare their results. The metrics used to evaluate and compare the ML algorithms were: accuracy, precision, sensitivity, specificity, F1 score, and AUC (Table 2; Figure 4). The metrics were calculated using the following equations:

$$\text{Accuracy}=\left(\mathrm{TP}+\mathrm{TN}\right)/\left(\mathrm{TP}+\mathrm{FP}+\mathrm{TN}+\mathrm{FN}\right)$$

$$\text{Precision}=\mathrm{TP}/\left(\mathrm{TP}+\mathrm{FP}\right)$$

$$\text{Sensitivity}=\mathrm{TP}/\left(\mathrm{TP}+\mathrm{FN}\right)$$

$$\text{Specificity}=\mathrm{TN}/\left(\mathrm{TN}+\mathrm{FP}\right)$$

$$F1-\text{score}=2\times \mathrm{TP}/\left(2\times \mathrm{TP}+\mathrm{FP}+\mathrm{FN}\right)$$

**TABLE 2:** Performance of the machine learning algorithms for men.

Algorithm	Accuracy	Precision	Sensitivity	Specificity	F1 score	AUC
LR	0.74 (0.67–0.81)	0.15 (0.12–0.18)	0.51 (0.44–0.58)	0.76 (0.69–0.83)	0.23 (0.18–0.28)	0.68 (0.61–0.75)
SVM	0.70 (0.63–0.77)	0.10 (0.07–0.13)	0.30 (0.25–0.35)	0.73 (0.66–0.80)	0.13 (0.10–0.16)	0.58 (0.51–0.65)
RF	0.91 (0.86–0.96)	0.30 (0.25–0.35)	0.27 (0.22–0.32)	0.96 (0.93–0.99)	0.31 (0.25–0.37)	0.73 (0.67–0.80)
KNN	0.63 (0.56–0.70)	0.11 (0.080–0.14)	0.51 (0.44–0.58)	0.64 (0.57–0.71)	0.18 (0.14–0.22)	0.61 (0.54–0.68)
GBM	0.92 (0.88–0.96)	0.53 (0.46–0.60)	0.24 (0.19–0.29)	0.98 (0.96–1.0)	0.33 (0.27–0.39)	0.75 (0.69–0.82)
XGB	0.87 (0.82–0.92)	0.26 (0.21–0.31)	0.32 (0.26–0.38)	0.92 (0.88–0.96)	0.28 (0.23–0.33)	0.72 (0.65–0.79)
BAG	0.90 (0.85–0.95)	0.32 (0.26–0.38)	0.25 (0.20–0.30)	0.96 (0.93–0.99)	0.29 (0.24–0.34)	0.73 (0.66–0.80)

Abbreviations: AUC, Area Under the Curve; BAG, bagging classifier; XGB, extreme gradient boosting; GBM, gradient boosting machine; KNN, K‐Nearest Neighbours; LR, logistic regression; RF, random forest; SVM, support vector machine.

Here, TP represents the true positive rate, TN the true negative rate, FP the false positive rate, and FN the false negative rate.

### 2.9 Model interpretation

**FIGURE 5:**
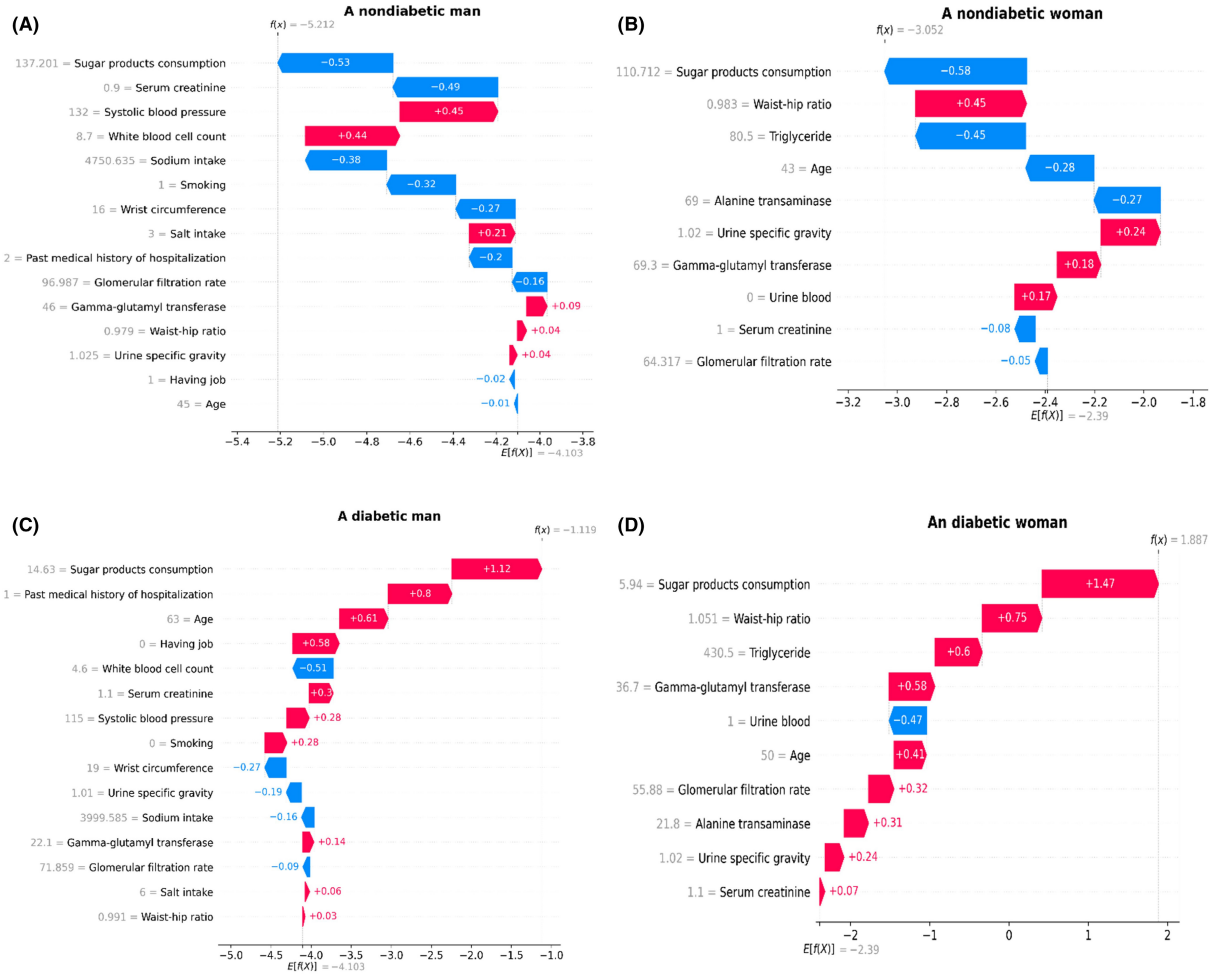
(A–D) The SHAP waterfall plot for four selected patients in the GBM model. GBM, gradient boosting machine; SHAP, SHapley Additive exPlanations.

The SHapley Additive exPlanations (SHAP) analysis was employed to gain insights into the GBM model. Specifically, SHAP values were computed for the top features (Figure 2A,B). Additionally, two randomly selected cases with different outcomes from each group were chosen to serve as examples, demonstrating the practical functioning of the GBM model (Figure 5).

## 3 RESULTS

### 3.1 Descriptive analyses

A total of 10,112 participants were included in this study. As shown in Table 1, 1,246 and 8866 participants were diagnosed with and without T2DM, respectively. 4566 (45%) of the participants were males and 5546 (55%) were females. After the feature selection, the top 15 features were selected to train the models for males and the top 10 features were selected for females. The results of the descriptive analysis are shown in Table 1.

Individuals with T2DM had a greater average age than those without T2DM (53.61 vs. 47.93). Women with T2DM had a higher percentage than men with T2DM (16.4 vs. 7.7). Individuals with T2DM were more likely to be smokers (6.7 vs. 93.3). Individuals with T2DM compared to those without had greater median SBP (117 vs. 110 mmHg). In Individuals with and without T2DM, the average of WRR, WC, WBC, triglyceride, ALT, GGT and serum creatinine were higher, which were 0.96 versus 0.92, 16,80 versus 16.71 (cm), 6.70 versus 6.43 (×10^9^/L), 152.12 versus 129.02 (mg/dL), 25.35 versus 23.17 (U/L), 27.55 versus 22.18 (U/L) and 0.99 versus 0.98 (mg/dL), respectively. Individuals with T2DM compared to those without had lower glomerular filtration rate, salt intake, sugar products consumption, and sodium intake, which were 70.08 versus 76.44, 3.69 versus 4.10, 35.44 versus 58.51 (g/day) and 4639 versus 4778 (mg/day), respectively. In the samples, the number of persons with positive urine blood was lower (282 vs. 2647). On average, individuals with T2DM had greater urine specific gravity (1.0206 vs. 1.0205). Individuals with T2DM had less medical history of hospitalization and jobs, which were 8.3 versus 91.7 and 7.9 versus 92.1, respectively.

Of the 13 variables found to have statistically significant differences, two variables, serum creatinine (p = .054) and urine specific gravity (p = .843), did not demonstrate statistical significance based on outcome classification.

### 3.2 Performance comparison of ML models

Tables 2 and 3 present the performance of the ML models based on various metrics for males and females, respectively. The final decision and determination of the best ML model were made by considering the AUC and F1‐score metrics.

**TABLE 3:** Performance of the machine learning algorithms for women.

Algorithm	Accuracy	Precision	Sensitivity	Specificity	F1 score	AUC
LR	0.68 (0.63–0.73)	0.28 (0.24–0.32)	0.64 (0.59–0.69)	0.69 (0.64–0.74)	0.39 (0.35–0.43)	0.71 (0.67–0.76)
SVM	0.68 (0.63–0.73)	0.29 (0.25–0.33)	0.69 (0.64–0.74)	0.68 (0.63–0.73)	0.41 (0.37–0.45)	0.75 (0.71–0.79)
RF	0.82 (0.78–0.86)	0.44 (0.40–0.48)	0.41 (0.37–0.45)	0.90 (0.87–0.93)	0.42 (0.38–0.46)	0.75 (0.71–0.80)
KNN	0.66 (0.61–0.71)	0.27 (0.23–0.31)	0.68 (0.63–0.73)	0.66 (0.61–0.71)	0.39 (0.35–0.43)	0.73 (0.68–0.77)
GBM	0.83 (0.79–0.87)	0.49 (0.44–0.54)	0.38 (0.34–0.42)	0.92 (0.89–0.95)	0.42 (0.38–0.46)	0.76 (0.71–0.80)
XGB	0.83 (0.79–0.87)	0.55 (0.50–0.60)	0.34 (0.30–0.38)	0.92 (0.89–0.95)	0.39 (0.35–0.43)	0.76 (0.72–0.80)
BAG	0.82 (0.78–0.86)	0.40 (0.36–0.44)	0.37 (0.33–0.41)	0.90 (0.87–0.93)	0.40 (0.36–0.44)	0.73 (0.69–0.77)

Abbreviations: AUC, Area Under the Curve; BAG, bagging classifier; XGB, extreme gradient boosting; GBM, gradient boosting machine; KNN, K‐Nearest Neighbours; LR, logistic regression; RF, random forest; SVM, support vector machine.

#### 3.2.1 Males

Table 2 displays the performance of the ML models for males. The GBM had the highest AUC (0.75). The AUCs of the other models were as follows: 0.73 for RF, 0.73 for BAG, 0.72 for XGB, 0.68 for LR, 0.61 for KNN, and 0.58 for SVM (Figure 4A). The GBM model also achieved the highest F1 score at 0.33, while the SVM model had the lowest F1‐score, 0.13. The F1 scores for the other models were as follows: RF (0.31), BAG (0.29), XGB (0.28), LR (0.23), and KNN (0.18). Consequently, based on AUC and F1‐score, the GBM model was selected as the best‐performing model. Additional metrics for the models are provided in Table 2.

#### 3.2.2 Females

Table 3 provides an overview of the performance of the ML models for females. All models demonstrated acceptable AUC values, with GBM and XGB achieving the highest at 0.76. The AUCs for the remaining models were: SVM (0.75), RF (0.75), BAG (0.73), KNN (0.73), and LR (0.71) (Figure 4B). For F1 score, both RF and GBM models attained the highest value of 0.42, while LR, KNN, and XGB had the lowest F1 score at 0.39. The F1 scores for the other models were 0.41 for SVM and 0.40 for BAG. Consequently, the GBM model was also chosen as the best‐performing model for females.

### 3.3 Interpretation of the best model

#### 3.3.1 Males

**FIGURE 6:**
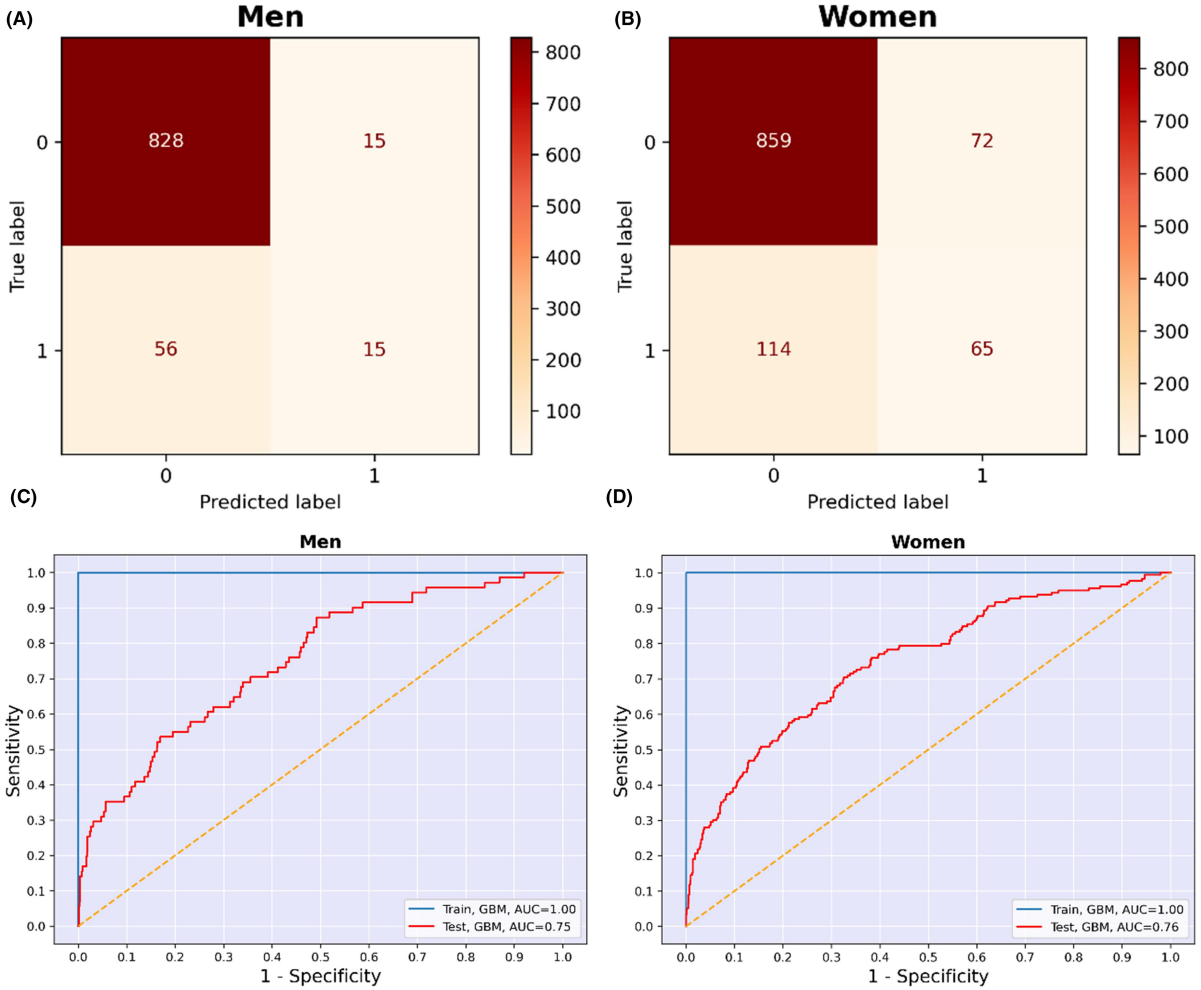
(A, B) Confusion matrix of the GBM model for identification of having T2DM. (C, D) Receiver operating characteristic curves of the GBM model for identification of having T2DM. GBM, gradient boosting machine; ROC, receiver operating characteristic.

Figure 6A presents the confusion matrix for the GBM model, showcasing its performance in identifying individuals with T2DM. Notably, the highest number of observations falls under true negatives (828), while true positives and false positives had the lowest number of observations (15). Moreover, Figure 6C illustrates the AUC of the GBM model for both training and test data (1.0 vs. 0.75, respectively).

The GBM model was utilized to identify the top 15 features. Figure 2A displays the SHAP values for these features. Age emerged as the most influential feature in accurately identifying individuals with T2DM. Following age, the subsequent significant features were sugar product consumption, past medical history of hospitalization, and serum creatinine. SBP and salt intake were ranked fifth and sixth, respectively.

Figure 5A presents the SHAP waterfall plot for two randomly selected men with different outcomes from the GBM model. The y‐axis indicates the input features in descending order of significance. The model output for an individual is represented as f(x). If f(x) surpasses e[f(x)], the participant has a higher probability of having T2DM compared to the background population. Each arrow signifies how a specific feature either increases (red) or decreases (blue) the participant's T2DM risk. For example, sugar product consumption of 137 g/day decreases the probability of having T2DM for case A (without T2DM), but sugar consumption of 14 g/day increases the probability of having T2DM for case C (without T2DM). The grey text before the feature names shows the value of each feature for each case.

#### 3.3.2 Females

Figure 5B presents the confusion matrix of the GBM model for the performance of each model in identifying individuals with T2DM. True negatives had the highest number of observations (859), while true positives had the lowest (65). In addition, Figure 6D shows the AUC of the GBM model for train and test data (1.0 vs. 0.76, respectively).

The GBM model was utilized to identify the top 10 features. Figure 2B displays the SHAP values for these features. Sugar consumption was the most accurate feature in identifying individuals with T2DM. The other important features were urine blood, age, WHR, ALT, and serum creatinine, respectively.

Figure 5 displays the SHAP waterfall plot for two randomly selected women with different outcomes from the GBM model. Sugar product consumption of 110 g/day reduces the probability of having T2DM for case B (without T2DM), but sugar product consumption of 5.94 g/day increases the probability of having T2DM for case D (without T2DM).

## 4 DISCUSSION

Our study aligns with several previously published studies investigating the application of ML algorithms for T2DM screening, risk stratification, prediction, and prognostic evaluation.^14^, ^15^, ^16^, ^17^, ^18^, ^19^ To the best of our knowledge, this study is the first to explore factors associated with T2DM while assessing the performance of ML models using cross‐sectional data from an Iranian population. Our findings highlight the superiority of the GBM model in T2DM screening within a south Iranian population. According to the GBM model, key associated factors for T2DM included sugar consumption, urine blood, and age in females, as well as age, sugar consumption, and a history of hospitalization in males.

ML represents a pivotal technique for translating health‐related data into practical knowledge. Implementation of such knowledge and expertise will advance clinical practice.^14^ In this regard, several systematic reviews and meta‐analyses are available.^15^, ^16^, ^17^, ^18^, ^19^, ^20^, ^21^ In recent years, ML models have gained significant attention for their potential in T2DM prediction, diagnosis, and management. Notably, in the studies by Abhari et al. and Tan et al., KNN, NVM, and NB were the most utilized ML models for T2DM data.^15^, ^19^

We analysed the performance of each ML model separately for males and females in our study. Our findings indicated that the overall AUC of ML models was between 67 and 80 for females and 51 and 82 for males. These results are similar to the previous high‐grade evidence.^15^ Sugar consumption and age were the shared variables among the three highest variables based on their SHAP value between both genders indicating the strong impact of these features on the model's performance. In addition, we found that urine blood and prior history of hospitalization were among the three highest variables regarding their SHAP value in females and males, respectively. These findings suggest the implementation of such variables in future models and designing potential data registries to consider these variables.

Previous studies have identified RF, SVM, KNN, and GBM as the optimal ML models for T2DM prediction.^15^, ^16^ While GBM demonstrated the best performance on our dataset, the present study yielded similar results, given the minor differences observed between the performances of other models. In this study, SVM, KNN, and RF showed an average AUC of 0.75, 0.73, and 0.75 for females, respectively. On the other hand, in males, these ML models showed an average AUC of 0.58, 0.61, and 0.73 for SVM, KNN and RF, respectively. Therefore, we can conclude that this study confirms the previous findings. Another crucial aspect, in addition to evaluating the performance of the ML models as conducted in this study, that should be taken into account when determining the optimal ML model is the assessment of potential financial benefits associated with each model. This consideration is recommended for exploration in future studies.

Our findings indicated that ML models were significantly more specific than sensitive in the FACS population. Therefore, regarding the clinical applications models could be useful as initial screening tools due to their high specificity, but additional testing (such as conventional T2DM detection, supervised by a medical team) may be required to confirm the diagnosis due to the potential for false negative results. However, the findings are inconsistent with previous research, as evidenced by Zanelli et al.,^21^ which includes biosignals such as photoplethysmography and electrocardiography as features yielded similar sensitivity and specificity. Consequently, future studies should assess other possible effective features (such as biosignals), additional confirmatory tests, or the need to consider other diagnostic criteria in conjunction with the ML results to improve the sensitivity while maintaining the specificity.

Due to the growing popularity of individualized medicine and the expanding size of health data, as well as the burden on healthcare centers, the integration of modern devices into the healthcare system has become imperative.^22^ ML models can continuously learn from data, resulting in improved performance as additional data is incorporated. Furthermore, they facilitate the development of user‐friendly applications that can identify high‐risk patients, reduce system overload, optimize budgets and finances and enhance the overall quality of healthcare. The mentioned reasons are particularly relevant when considering T2DM, a chronic condition that can impact various bodily systems, have significant financial implications for patients and public health, and is efficiently manageable with appropriate interventions. Timely identification of individuals at high risk is crucial for effective disease control and prevention of complications.

### 4.1 Strengths and limitations

This study is the first to utilize ML models for screening T2DM within an Iranian population, incorporating a diverse set of baseline variables. These variables included sociodemographic, anthropometric, clinical and paraclinical factors, as correlates for having T2DM, all within a significantly large sample size. Notably, the study distinguishes itself by utilizing seven diverse ML models for comparison, constituting one of the most extensive sets of ML models employed in a single study, as suggested by previous systematic reviews.

The present study had some limitations, primarily due to its cross‐sectional design, which hinders longitudinal follow‐up. The absence of a time component may introduce some biases to the study. Additionally, while internal validation is a common practice in studies utilizing the performance of the ML models, ML studies in T2DM often lack external validation,^17^ a limitation shared by our study. The absence of external validation brings uncertainty to the results and restricts the generalizability of the findings. Moreover, it's important to note that the findings of our study were based on data from a rural region in Iran, which may not be entirely representative of other populations in the country. Furthermore, it's crucial to acknowledge that the data under investigation were collected approximately 6–8 years earlier. This temporal gap raises the possibility that the population's characteristics and risk factors may have evolved over time. Additionally, caution is advised when interpreting findings from ML models applied to biological data, as they inherently exhibit limitations in drawing causal inferences.^23^ Therefore, any conclusions drawn from the findings of the current study must be approached with caution, taking into account the mentioned limitations.

## AUTHOR CONTRIBUTIONS

**Hanieh Karmand:** Conceptualization (supporting); investigation (equal); writing – original draft (equal). **Aref Andishgar:** Conceptualization (supporting); formal analysis (equal); investigation (equal); software (equal); visualization (equal). **Reza Tabrizi:** Conceptualization (equal); formal analysis (equal); project administration (equal); writing – review and editing (equal). **Alireza Sadeghi:** Conceptualization (equal); data curation (equal); formal analysis (equal); investigation (equal); writing – original draft (equal); writing – review and editing (equal). **Babak Pezeshki:** Investigation (equal); methodology (equal); writing – original draft (equal). **Mahdi Ravankhah:** Investigation (equal); methodology (equal); writing – original draft (equal). **Erfan Taherifard:** Conceptualization (equal); formal analysis (equal); methodology (equal); writing – original draft (equal); writing – review and editing (equal). **Fariba Ahmadizar:** Conceptualization (equal); data curation (equal); formal analysis (equal); methodology (equal); supervision (equal); writing – review and editing (equal).

## FUNDING INFORMATION

This research received no specific grant.

## CONFLICT OF INTEREST STATEMENT

None.

## ETHICS STATEMENT

The study protocol was approved by the regional and national research ethics committees (equivalent to institutional review boards) of Fasa University of Medical Sciences (IR.FUMS.REC.1402.039). All participants were asked to sign a written informed consent approved by the research ethics committee. All the participants' information was collected from the system, with all names erased.

## CONSENT TO PUBLISH

All participants were asked to sign a written informed consent approved by the research ethics committee.

## Supporting information


Table S1.
Table S2.
Table S3.
**Table S4**.

## Data Availability

Data are available on request.
