# Supplementary material for: Machine‐learning algorithms in screening for type 2 diabetes mellitus: Data from Fasa Adults Cohort Study
Source: Endocrinol Diabetes Metab. 2024 Feb 27;7(2):e00472. doi: 10.1002/edm2.472 (PMC10897867; doi:10.1002/edm2.472)
Supplement: Supplementary file 1 — Table S1. Table S2. Table S3. Table S4. [file EDM2-7-e00472-s001.docx]

Machine-Learning Algorithms in Screening for Type 2 Diabetes Mellitus: Data from Fasa Adults Cohort Study

Supplementary Material

| **Content** | **Page Number** |
| --- | --- |
| **Supplementary Table 1**. Overview of shared characteristics and clinical features of participants | 2 |
| **Supplementary Table 2**. Overview of male- and female-specific variables | 4 |
| **Supplementary Table 3.** Identification of optimal hyper-parameter values for each algorithm after hyper-parameter tuning for men | 5 |
| **Supplementary Table 4**. Identification of optimal hyper-parameter values for each algorithm after hyper-parameter tuning for women | 6 |

| **Supplementary Table 1.** Overview of shared characteristics and clinical features of participants | |
| --- | --- |
| **Non-laboratory variables** | |
| Age | History of Mouth Aphthous Lesion |
| Ethnicity | History of Genital Aphthous Lesion |
| Marital status | History of Rheumatoid Arthritis |
| Education years | History of Surgery |
| History of Diabetes | History of Hospitalization |
| History of Hypertension | History of Transfusion |
| History of Cardiac Disease | Job status |
| History of Myocardial Infarction | Sleeping Pills Use |
| History of Stroke | Sleep Duration |
| History of Renal Failure | Diastolic Blood pressure |
| History of Fatty Liver | Systolic Blood pressure |
| History of Hepatitis B | Pulse Rate |
| History of Hepatitis C | Height |
| History of Chronic Lung Disease | Weight |
| History of Thyroid Disease | Waist Circumference |
| History of Kidney Stone | Hip Circumference |
| History of Gallstone | Wrist Circumference |
| History of Rheumatic Disease | Waist-to-hip ratio |
| History of Skin Cancer | Waist-to-height ratio |
| History of Stomach Cancer | Body Mass Index |
| History of Colorectal Cancer | Smoking |
| History of Bladder Cancer | Use Alcohol |
| History of Esophagus Cancer | Family History of Hypertension |
| History of Lung Cancer | Family History of Cardiac Disease |
| History of Brain and CNS Cancer | Family History of Myocardial Infarction |
| History of Epilepsy | Family History of Stroke |
| History of Chronic Headaches | Family History of Stomach Cancer |
| History of Depression | Family History of Colorectal Cancer |
| History of Psychiatric Disorder | Family History of Skin Cancer |
| History of Laryngeal Cancer | Family History of Bladder Cancer |
| History of Tongue Cancer | Family History of Lung Cancer |
| History of Lupus | Family History of Brain CNS Cancer |
| History of Multiple Sclerosis | Family History of Alzheimer |
| History of Cardiovascular Diseases | Family History of Pelvic Femoral Fracture |
| History of Sternal Irritation | Family History of Laryngeal Cancer |
| History of Swelling | Family History of Tongue Cancer |
| History of Urine Color Changes | Family History of Lupus |
| History of Heartburn | Family History of Multiple Sclerosis |
| History of Food Regurgitation | Family History of Prostate Cancer |
| History of GERD | Family History of Breast Cancer |
| History of Blood in Stool | Family History of Uterine Cancer |
| History of Weight Loss | Family History of Ovarian Cancer |
| History of Jaundice | Metabolic Equivalent of Task (MET) |
| History of Shortness of Breath | Socioeconomic Status |
| History of Gait Problem | Total Lipid Fat Intake |
| History of Faint | Total Carbohydrate Intake |
| History of Thought Disorder | Total Fiber Intake |
| History of Visual Impairment | Calcium Intake |
| History of Muscle Weakness | Cholesterol Intake |
| History of Movement Disorder | Total Trans Fatty Acid Intake |
| History of Numbness | Iron Intake |

| History of Recurring Headaches | Sodium Intake |
| --- | --- |
| History of Dizziness | Caffeine Intake |
| History of Tinnitus | Bread and Grain Consumption |
| History of Five-Year Fracture | Nuts Consumption |
| History of Fracture Ever | Meats Consumption |
| History of Hip Femoral Fracture | Dairy Product Consumption |
| History of Osteoporosis | Vegetables Consumption |
| History of Back Pain | Fruits Consumption |
| History of Joint pain | Sugar Products Consumption |
| History of Joint Stiffness | Salt Consumption |
| **Laboratory variables** | |
| White Blood Cell (WBC) | Aspartate Aminotransferase (AST) |
| Red Blood Cell (RBC) | Alanine Transaminase (ALT) |
| Hemoglobin | Alkaline Phosphatase |
| Hematocrit | HDL-C |
| Mean Corpuscular Volume (MCV) | LDL-C |
| Mean Corpuscular Hemoglobin (MCH) | Gamma-glutamyl transferase (GGT) |
| Mean Corpuscular Hemoglobin Concentration (MCHC) | Urine Specific Gravity |
| Platelet | Urine Nitrite |
| Lymphocyte | Urine Bilirubin |
| Monocyte | Urine Urobilinogen |
| Granulocyte | Urine Protein |
| Blood Urea Nitrogen (BUN) | Urine Glucose |
| Creatinine Level | Urine Blood |
| Triglyceride Level | Urine Crystal |
| Cholesterol Level | Urine Ketones |
| Glomerular Filtration Rate |  |

| **Supplementary Table 2.** Overview of male- and female-specific variables | |
| --- | --- |
| **Specific variables for male** | |
| History of Prostate Cancer |  |
| **Specific variables for female** | |
| History of Breast Cancer | History of Pregnancy Hypertension |
| History of Uterine Cancer | History of Pregnancy Diabetes |
| History of Ovarian Cancer |  |
| Number of Pregnancies |  |
| History of Stillbirth |  |
| Number of Abortions |  |

| **Supplementary Table 3.** Identification of optimal hyper-parameter values for each algorithm after hyper- parameter tuning for men | |
| --- | --- |
| Algorithm | Optimal hyper-parameters |
| LR | C=100, solver='liblinear', multi_class = ‘auto’, penalty = 12 |
| SVM | C = 1, gamma = 'scale', kernel= 'rbf', probability=True |
| RF | max_features = 'sqrt', n_estimators = 100 |
| KNN | metric= 'euclidean', n_neighbors= 11, weights= 'uniform' |
| GBM | learning_rate= 0.01, max_depth=9, n_estimators= 1000, subsample= 0.5 |
| XGB | n_estimators=180, colsample_bytree= 0.5, gamma= 1, max_depth=1, min_child_weight=10, reg_alpha=1, reg_lambda=1 |
| BAG | n_estimators= 100 |
| Abbreviations: LR; Logistic Regression, SVM; Support Vector Machine, RF; Random Forest, KNN; K-Nearest Neighbors, GBM; Gradient Boosting Machine, XGB; Extreme Gradient Boosting, BAG; Bagging classifier | |

| **Supplementary Table 4.** Identification of optimal hyper-parameter values for each algorithm after hyper- parameter tuning for women | |
| --- | --- |
| Algorithm | Optimal hyper-parameters |
| LR | C=10, solver='liblinear', multi_class = ‘auto’, penalty = 12 |
| SVM | C = 0.1, gamma = 'scale', kernel= 'rbf', probability=True |
| RF | max_features = 'sqrt', n_estimators = 1000 |
| KNN | metric= 'euclidean', n_neighbors= 15, weights= 'uniform' |
| GBM | learning_rate= 0.01, max_depth=3, n_estimators= 1000, subsample= 1 |
| XGB | n_estimators=180, colsample_bytree= 0.5, gamma= 9, max_depth=3, min_child_weight=0, reg_alpha=1, reg_lambda=1 |
| BAG | n_estimators= 1000 |
| Abbreviations: LR; Logistic Regression, SVM; Support Vector Machine, RF; Random Forest, KNN; K-Nearest Neighbors, GBM; Gradient Boosting Machine, XGB; Extreme Gradient Boosting, BAG; Bagging classifier | |
